# Multi-objective learning and explanation for stroke risk assessment in Shanxi province

**DOI:** 10.1038/s41598-022-26595-z

**Published:** 2022-12-26

**Authors:** Jing Ma, Yiyang Sun, Junjie Liu, Huaxiong Huang, Xiaoshuang Zhou, Shixin Xu

**Affiliations:** 1 Research Center for Mathematics, Beijing Normal University, Zhuhai, 519087 China; 2 Laboratory of Mathematics and Complex Systems (Ministry of Education), School of Mathematical Sciences, Beijing Normal University, Beijing, 100875 China; 3 Global Health Research Center, Data Science Research Center, Duke Kunshan University, 8 Duke Ave, Kunshan, Jiangsu, China; 4 BNU-HKBU United International College, Zhuhai, China; 5 Department of Mathematics and Statistics, York University, Toronto, ON Canada; 6 Department of Nephrology, Shanxi Provincial People’s Hospital, Taiyuan, Shanxi China

**Keywords:** Scientific data, Risk factors

## Abstract

Stroke is the leading cause of death in China (Zhou et al. in The Lancet, 2019). A dataset from Shanxi Province is analyzed to predict the risk of patients at four states (low/medium/high/attack) and to estimate transition probabilities between various states via a SHAP DeepExplainer. To handle the issues related to an imbalanced sample set, the quadratic interactive deep model (QIDeep) was first proposed by flexible selection and appending of quadratic interactive features. The experimental results showed that the QIDeep model with 3 interactive features achieved the state-of-the-art accuracy 83.33%(95% CI (83.14%; 83.52%)). Blood pressure, physical inactivity, smoking, weight, and total cholesterol are the top five most important features. For the sake of high recall in the attack state, stroke occurrence prediction is considered an auxiliary objective in multi-objective learning. The prediction accuracy was improved, while the recall of the attack state was increased by 17.79% (to 82.06%) compared to QIDeep (from 71.49%) with the same features. The prediction model and analysis tool in this paper provided not only a prediction method but also an attribution explanation of the risk states and transition direction of each patient, a valuable tool for doctors to analyze and diagnose the disease.

## Introduction

Stroke is the leading cause of death in China, and worldwide the second leading cause of death in people older than 60 years and the fifth leading cause of death among those aged 15 to 59 years old^1^. More than two-thirds of stroke deaths occur in developing countries, almost one-third occurring in China^2^. By 2017, stroke became one of the top three causes of death^3^, and accounted for 1.57 million deaths in 2018^4^. The rising number of stroke patients has put immense pressure on the public health system.

Stroke is a preventable disease. A certain number of potential risk factors, such as age, systolic blood pressure, and smoking, have been identified^5^, which provided some useful information for the general public. However, better algorithms are needed to improve the accuracy of predicting the risk of stroke and increase the effectiveness of preventive measures^6^. Traditional statistical methods, such as the Framingham Stroke Risk Profile (FSRP)^7^, new FSRP^8^, and QStroke^9^, aim to predict either 10-year or 5-year risk of stroke^10^. But the performance of the algorithms depends heavily on the preselected features.

Machine learning (ML) techniques comprise a set of powerful algorithms that are capable of modeling complex and hidden relationships between a multitude of clinical variables and the desired clinical outcome without stringent statistical assumptions. There is a growing interest in the application of machine learning techniques to address clinical problems^11–14^. Unlike the steady growth in the application of ML methods in other industries, the utilization of the ML approach, especially the use of deep learning in electronic health records (EHRs) appears only recently. A high-performance DNN model^15^ was developed to predict 3-year and 8-year stroke occurrences based on a large EHR dataset. It was also demonstrated in^16–18^ that DNN performed better than logistic regression (LR) and random forest (RF) for predicting the long-term outcome of stroke occurrence. Furthermore, the combination of real-time electromyography biosignals data^19^ and long short-term memory (LSTM) algorithm with a balance the memory ratio between records (long-term) and real-time data (short-term) has showed some promises. However, most of these methods are mostly focused on two-states classification, i.e., Stroke/Non-stroke.

Based on the census data in both communities and hospitals from Shanxi Province, a model was proposed in^20^ for predicting three risk states (low/medium/high), and stroke risk factors were ranked within the 8 + 2 group of main risk factors by the China National Stroke Prevention Project (CSPP), with hypertension, physical inactivity (lack of sports), and obesity as the top three in Shanxi.

In this paper, we use the 8 + 2 main risk factors to identify an additional risk state. The attack (most urgent) state is introduced to represent patients who have experienced at least one stroke, motivated by many studies that have considered stroke occurrence. Furthermore, we provide predictions and attributions of the current risk state and transitional probabilities for each patient. For example, when one patient is in a high-risk state, abnormal glycosylated hemoglobin and other factors make it possible for the patient to move into the attack state. This prompts us to add the attack state to the other three risk states considered in^20^.

The main contributions of this paper are described as follows.

- A DNN model with four-classification multilayer perceptrons (MLPs) is used a baseline for identifying risk states against the state-of-the-art tree models in healthcare (in “Experiment setup”). SHAP analysis tool is applied to obtain the determinants of each patient’s current risk state and their transitional probabilities.
- A quadratic interactive deep model (QIDeep) is incorporated for improving the accuracy of the base DNN model by appending selective quadratic interactive features. Compared with the baseline DNN model, the mean of the recalls in all four states increased from $80.29\%$ to $81.16\%$, the precision increased from $82.10\%$ to $83.33\%$ and the number of iterations decreased from 48 to 29 with three additional features with order-2 interactions in the QIDeep model.
- A multi-objective model is developed to improve the recall rate for the most urgent attack state. The effects of sample imbalance were reduced as well as more accurate prediction of the attack state. Specifically, the accuracy increased from $80.58\%$ to $84.45\%$ and the recall improved from $67.93\%$ to $84.83\%$ compared to the QIDeep model with three order-2 features.

The reminder of the paper is organized as follows. “Methods” section describes the proposed stroke risk prediction model and optimization methods, and results are presented in “Results” section, where implementation issues of the proposed method are also discussed. Concluding remarks are provided in “Conclusions” section.

## Methods

**Figure 1:**
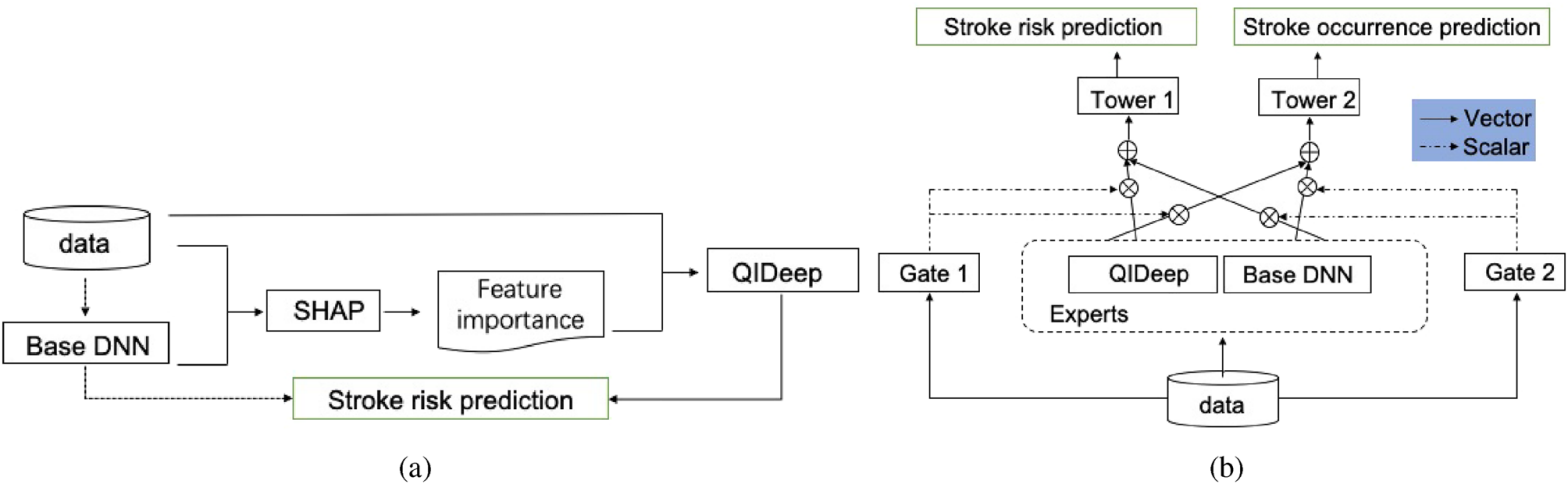
The overall workflow, the tasks are emphasized by green boxes: (**a**) produces two stroke risk predictions. The first process is described by the dashed line that the base DNN is trained to predict stroke risks. The solid line depicts the process of QIDeep. The data and the trained based DNN model are fed into SHAP to compute feature importance. Then, QIDeep swallows the data and refers to the feature importance to improve the performance of stroke risk predictions. (**b**) is a two-objective learning model. QIDeep and base DNN are experts to extract information from the data. Their contributions to each task are represented by aligned vectors and summarized by the scalers returned by Gate functions. The Towers translate the weighted contributions to specific tasks which predict the two objectives simultaneously.

**Figure 2:**
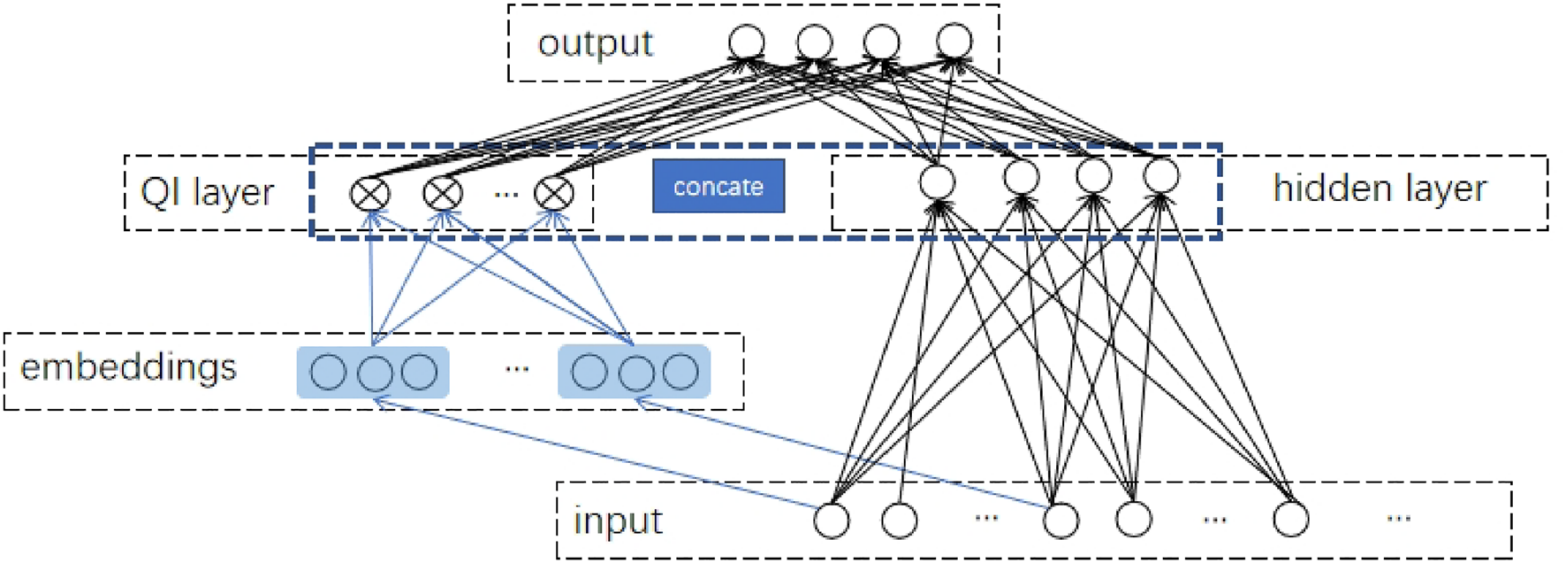
Illustration of QIDeep.

In this section, two models and a model interpretation method are introduced for stroke risk prediction and intervention. The workflow of the aforementioned contents is demonstrated in Fig. 1. Figure 1a describes the process of QIDeep, which improves the performance of the stroke risk prediction from the base DNN. The model interpretation method SHAP takes the data and the trained DNN and returns feature importance. We select top-k important features to construct the order-2 features that are being fed to QIDeep (shown in Fig. 2) along with the original data. The multi-gate mixture-of-experts (MMOE) model, which further improves stroke risk prediction, is depicted in Fig. 1b. It takes the QIDeep and the base DNN as experts that contribute to both the stroke risk prediction and the stroke occurrence prediction, which serves as the auxiliary objective. In this section, we first briefly review the material and tools that are utilized. By considering the order-2 interactions, we propose QIDeep to improve the performance for stroke risk prediction. Last but not least, stroke occurrence prediction is considered an auxiliary objective to benefit from the multi-gate mixture-of-experts (MMOE) model to achieve a higher recall.

### Data

The China National Stroke Prevention Project (CSPP) was launched by the Chinese government as the key national action on stroke screening and intervention since 2011. The dataset applied in this study consists of survey data and laboratory data from Shanxi Province between 2017 and 2020. Adults who agreed to complete the CSPP survey and had resided in Shanxi Province for at least 6 months were invited to participate in the screening process. All participants were registered at the local government office. The study was conducted according to the Declaration of Helsinki. The original data included 27,583 residents from 2017 to 2020 and 2,000 hospitalized stroke-attacked patients in 2018. After cleaning, 34 features are employed, including sex, age, and other basic features; smoking, lack of exercise (physical inactivity), and other lifestyle features; and laboratory data such as blood pressure. Table 1 lists and explains some of the main features. After cleaning, the numbers of the four states (low:medium:high:attack) are 7221:5868:5475:1967. If the low-, medium- and high-risk data were merged into the non-stroke category, the sample ratio of stroke patients to non-stroke patients was nearly 1:10.

**Table 1:** Explanation of Selected Features.

Abbreviations	Feature name	Explanation
Arhm	Arrhythmia	1: YES. 0: NO
BMI	Body Mass Index	Normal range: 20–25
Edu	Education	1: Primary school and below. 2: Junior high school. 3: Technical secondary school/senior high school. 4: Junior college/undergraduate. 5: Master’s degree or above
Exs	Lck of exercise	1: YES. 0: NO
FA	Filling age	–
FBG	Fasting blood glucose	Normal range: 3.9–6.1 mmol/L
HbA1c	glycosylated hemoglobin	Normal range: 4–6%
Hcy	Homocysteine	Normal range: 5–15 $\mathrm {\mu mol/L}$
HDL-C	High density lipoprotein cholesterol	Normal range for female: 1.29–1.55 mmol/L; normal range for male: 1.16–1.42 mmol/L
HEH	History of hypertension	1: YES. 0: NO
HS	History of stroke	1: YES. 0: NO
Ht/Wt	Height/weight	–
LDBP	Left diastolic blood pressure	Normal range: 60–89 mmHg
LDL-C	Low density lipoprotein cholesterol	Normal range: < 3.37 mmol
LSBP	Left systolic blood pressure	Normal range: 80–140 mmHg $^{1}$
MV	Meat and vegetable	1: Equilibrium. 2: Partial vegetable. 3 Partial meat.
Ret	Retire	1: YES. 0: NO
Sm	Smoking	2: YES. 1: Quitting. 0: NO
TC	Total cholesterol	3–5.2 mmol/L
TG	Triglyceride	Normal range: 0.6–1.7mmol/L
Ys	years of smoking	Null for nonsmokers

$^1$ The normal range of LSBP is 80–140 mmHg and the optimal range is 80–120 mmHg.

### SHAP

Feature importance refers to techniques that assign a score to input features based on their usefulness in predicting a target variable. Traditional feature importance methods can only rank the importance of different features, without explaining how the feature affects the predictions (positively or negatively). Proposed by Shapley in 1953^21^, the Shapley method is the only attribution method that satisfies the four attributes of efficiency, symmetry, dummy, and additivity, which can be regarded as the definition of fair expenditure.

SHAP^22^ is a ”model interpretation” package developed by Python, which can interpret the output of most machine learning models. Inspired by Shapley, SHAP constructs an additive interpretation model, which interprets the predictive value of the model as the sum of the attribution values of each input feature. All features are regarded as ”contributors”, and the SHAP value is the average marginal contribution of the features to the output of the model.

Denote the *i*-th sample as $\varvec{x}_i$ and the *j*-th feature of the *i*-th sample as $x_{ij}$. Let $y_i$ be the predicted value of the model for this sample, and let $y_{base}$ represent the baseline output of the model. The following equation then holds:

1 $$\begin{aligned} y_i = y_{base}+f(x_{i1})+f(x_{i2})+\cdots +f(x_{ik}), \end{aligned}$$

where $f(x_{ij})$ is the SHAP value of $x_{ij}$. Intuitively, $f(x_{i1})$ is the contribution of the first feature in the *i*-th sample to the final predicted value $y_i$. The value $f(x_{i1})>0$ indicates that the feature improves the predicted value with a positive effect. On contrary, a negative value of $f(x_{i1})$ indicates that the feature has a negative effect.

### QIDeep

The main goal is to improve the model performance of predicting stroke risk with a small training dataset. Thus, popular methods combining all possible pairs of features, such as DeepFM, bring more computational burden and may not work well with the stroke risk assessment. The convolution methods are well known for their capacity to extract the local correlation and are popular in computer visions, recently, studies of implementing CNN to temporal sequences show the advantages over RNN^23^. However, the stroke census dataset is not able to ensure convergence if conducted by the aforementioned methods. Fortunately, the SHAP interaction values instruct the feature combination explicitly. To further improve the model’s accuracy and the ability to learn sophisticated feature interactions, a multiclassification model, referred to as the QIDeep, is proposed to predict stroke risk with a small dataset. The structure of QIDeep is shown in Fig. 2; it consists of two components: a QI component and a deep component. The deep component is a feed-forward neural network that is utilized to learn high-order feature interactions. The original input, which contains 34 categorical-continuous-mixed original features, enters the hidden layers for information extraction. Features with order-2 interactions are selected and input to the embedding layer. The latent vector $V_i$, which is obtained from the embedding layer, is fed into the QI component to model order-2 interactions to measure its impact on other features. The output probabilities of the four states are produced by *softmax* function with the concatenation of both components as the input,

2 $$\begin{aligned} y=softmax(y_{QI} \oplus y_{DNN}), \end{aligned}$$

where *y* is the predicted value, $y_{QI}$ is the output of the QI component, $\oplus$ means concatenate, and $y_{DNN}=softmax(W^oa^h+b^o)$ is the output of the deep component with weight $W^o$, bias $b^o$, of the output layer, and the output of the hidden layer $a^h$.

The QI component models pairwise feature interactions as the inner product of respective feature latent vectors. This component can more effectively capture order-2 feature interactions. This idea originates from DeepFM^24^, while direct usage of DeepFM may fail to converge due to a large number of parameters in our small dataset. As shown in Fig. 2, similar to factorization machines (FM)^25^, the output of the QI component is the number of inner product units:

3 $$\begin{aligned} y_{QI}=\sum _{i=1}^{n}\sum _{j=i+1}^{n}<V_i,V_j>x_{i}{x_{j}}=\frac{1}{2}\sum _{l=1}^{k}\left[ \left( \sum _{i=1}^{n}v_{i,l}x_i\right) ^2-\sum _{i=1}^{n}v_{i,l}^2x_i^2\right] , \end{aligned}$$

where *n* is the number of combination features in the QI layer, *k* is the length of the latent vector, $V_i$ is the latent vector representation of feature $x_i$, and $v_{i,l}$ is the value of feature *i* at the *l*-th position of the latent vector.

The main advantage of QIDeep is that by adding order-2 interactions into the multiclassification model, it could flexibly control the number of combined features, balancing the number of parameters and sample size to ensure model convergence with a small dataset.

### MMOE

The recall of the attack state is more important for clinical diagnosis since ideally every urgent case should be predicted correctly. MMOE model^26^, one of the most popular methods of multi-objective learning, is selected to further refine the attack state without obviously increasing the number of model parameters.

Stein’s paradox^27^ in statistics states that estimating the means of three or more Gaussian random variables using samples from all of them could yield better performance than estimating them separately. Multi-objective learning refers to learning and optimizing multiple tasks simultaneously through the benefit of common information and specific information among tasks.

As shown in Fig. 1b we apply the MMOE model to simultaneously optimize the predictions of both stroke occurrence and stroke risk. The intermediate layers of DNN and QIDeep are embedded as Expert1 and Expert2, respectively. Combined with weights produced by gates, we input the weighted sum of comprehensive opinions of experts to towers to learn the characteristics of each objective. Tower1 returns the stroke occurrence prediction, and Tower2 outputs the stroke risk prediction. We note that stroke occurrence prediction is considered an auxiliary objective to attract more attention to the attack state^28^.

By MMOE, both objectives can be achieved through common information and specific information. Concretely, in Fig. 1b, for objective $k\in \{1,2\}$, $h_k$ stands for the mapping to predict objective *k* ,

4 $$\begin{aligned} {h_k(x)=t^k(f^k(x)),} \end{aligned}$$

where $t^k$ is the *k*-th tower network, consists of multilayer full connections. The input of the tower, $f^k(x)=\sum _{i=1}^{n}g^k_i(x)f_i(x)$, is the weighted sum of each expert by the corresponding gate. It indicates that the gating networks of the *k*-th objective realize the selective utilization of experts by different weights. The *n* expert networks are denoted as $\{f_i\}_{i=1}^{n}$, and $g_i$ is the weight of the $i_{th}$ expert on the final decision that satisfies $\sum _{i=1}^{n}g_i(x)=1$. In the learning of different objectives, the effects of different experts’ opinions are adjusted through the gates.

The loss function is expressed as follows:

5 $$\begin{aligned} {L(h_1,h_2)=\sum _{i=1}^Nl_1(y_i,h_1(x_i))+\sum _{i=1}^Nl_2(z_i,h_2(x_i))}, \end{aligned}$$

where $h_1$ and $h_2$ are mappings to predict the stroke occurrence prediction and stroke risk prediction, respectively; $l_1$ is the binary cross entropy function, $l_2$ is the cross entropy function; and *y* and *z* are the labels of the stroke occurrence prediction and stroke risk prediction, respectively.

### Ethics declarations

The study was approved by the Ethics Review Committee of Shanxi Provincial people’s Hospital (No.223). The requirement of informed consent was waived by the Ethics Review Committee of Shanxi Provincial people’s Hospital.

## Results

In this section, we compare the performance between the proposed QIDeep model and several state-of-the-art models and show the proceeding improvement of the attack state by MMOE.

### Experiment setup

#### Parameter settings

As previously mentioned, we compare the multiclassification results among DNN, RF, QIDeep, and MMOE. Hence, we introduce the structures of these models.

For the RF model, we simply invoke the sklearn.ensemble.RandomForestClassifier.

For the DNN model, the input layer has 34 neurons connected to a hidden layer with 17 neurons. These two layers are fully connected with ReLU as the activation function followed by a dropout with a rate of 0.2. The output layer has 4 neurons.

For the QIDeep model depicted in Fig. 2, the deep component shares the same structure as the DNN model. By the sorted feature importance, the top 3 order-2 interactions to the top 7 order-2 interactions are selected to go through the QI component, where four is chosen as the embedding dimension. The outputs of the deep component and QI component are concatenated to feed forward to the output layer that contains four neurons.

For the MMOE model, to address the small dataset, we apply three measures to guarantee the convergence of MMOE. (1) remove the towers, and feed the result directly to the output layer. (2) decrease inputs from the top 34 features to the top 20 features; (3) Expert1 contains only one single hidden layer with 11 neurons, and Expert2 is a QIDeep network with the top 3 features for the QI component.

In the numerical experiments, we apply Adam with a learning rate of 0.01 to optimize the models, and the early stop mechanism with patience 20 is used to prevent overfitting.

#### Evaluation metrics

We use four evaluation metrics in our experiments:

- *Accuracy* the proportion of the number of correctly predicted samples to the total number of samples. Accuracy is the most intuitive indicator to measure the quality of the model.
- *Precision* the proportion of the number of positive samples correctly predicted to the number of positive samples predicted. The higher the precision, the more accurate the prediction.
- *Recall* the proportion of the number of positive samples correctly predicted to the number of all positive labels. The higher the recall, the more complete the result.
- *F1 score* $:=2\frac{\text {precision}\times \text {recall}}{\text {precision}+\text {recall}}$ is the harmonic mean of the precision and recall.

#### Model comparison

Presently, the state-of-the-art models of machine learning in healthcare are based on tree models^20,29,30^ and DNNs^15,16^. In the numerical experiments, we feed 34 features into the DNN model and RF model, and 20 features with 3 order-2 features into the QIDeep and MMOE models. We run the RF model 10 times, with the number of estimators increasing from 10 to 19, and the rest models are run 50 times, and expectation and standard variation are computed for the precision, recall, and f1 score. The numbers of the four states (low:medium:high:attack) for testing are 1, 096 : 881 : 813 : 290. The results are summarized in Table 2. RF works fairly well for the first three states (low/medium/high-risk). However, the imbalance of data causes poor recall of the attack state. The DNN model can predict the attack state better, while the results of the low-, medium- and high-risk models are slightly worse than those of the RF model. In clinical diagnosis, the ability to the prediction of the attack state is often more important, ideally correctly predicting every sample that is in the attack state. Hence, the DNN model, with a much higher recall of the attack state, is employed as a comparative model in subsequent stroke risk prediction experiments. This finding reveals that QIDeep works well with fewer features, and MMOE outperforms all the other models.

**Table 2:** Classification report of base DNN model, random forest (34 features) and QIDeep, MMOE (20 features).

Model	Risk state	Precision (%)	Recall (%)	F1 score (%)
Base DNN	Low	$82.82 \pm 0.083$	$89.20 \pm 3.143$	$85.86 \pm 1.495$
	Medium	$79.86\pm 1.549$	$77.91\pm 1.753$	$78.85\pm 0.872$
	High	$79.89\pm 2.852$	$76.03\pm 2.184$	$77.84\pm 1.92$
	Attack	$85.82\pm 2.507$	$78.01\pm 2.162$	$81.68\pm 1.145$
Random Forest	Low	$83.62\pm 0.320$	$96.80\pm 0.417$	$89.23\pm 0.333$
	Medium	$85.23\pm 0.850$	$86.30\pm 0.599$	$85.76\pm 0.714$
	High	$85.77\pm 1.076$	$77.08\pm 1.130$	$81.19\pm 1.073$
	Attack	$92.46\pm 0.616$	$\mathbf {60.10}\pm 0.951$	$72.85\pm 0.862$
QIDeep	Low	$82.55\pm 0.281$	$93.34\pm 0.610$	$87.61\pm 0.160$
	Medium	$81.84\pm 0.632$	$79.95\pm 0.054$	$80.88\pm 0.334$
	High	$79.77\pm 0.608$	$73.10\pm 1.256$	$76.29\pm 0.949$
	Attack	$88.43\pm 3.094$	$71.49\pm 1.875$	$78.99\pm 0.070$
MMOE	Low	$83.68\pm 0.738$	$93.80\pm 1.070$	$88.45\pm 0.524$
	Medium	$82.53\pm 1.282$	$81.12\pm 1.561$	$81.80\pm 0.687$
	High	$83.85\pm 1.832$	$73.59\pm 2.121$	$78.34\pm 0.873$
	Attack	$87.97\pm 2.056$	$82.06\pm 3.043$	$84.87\pm 1.944$

Significant values are in bold.

### Explanation of feature importance

**Figure 3:**
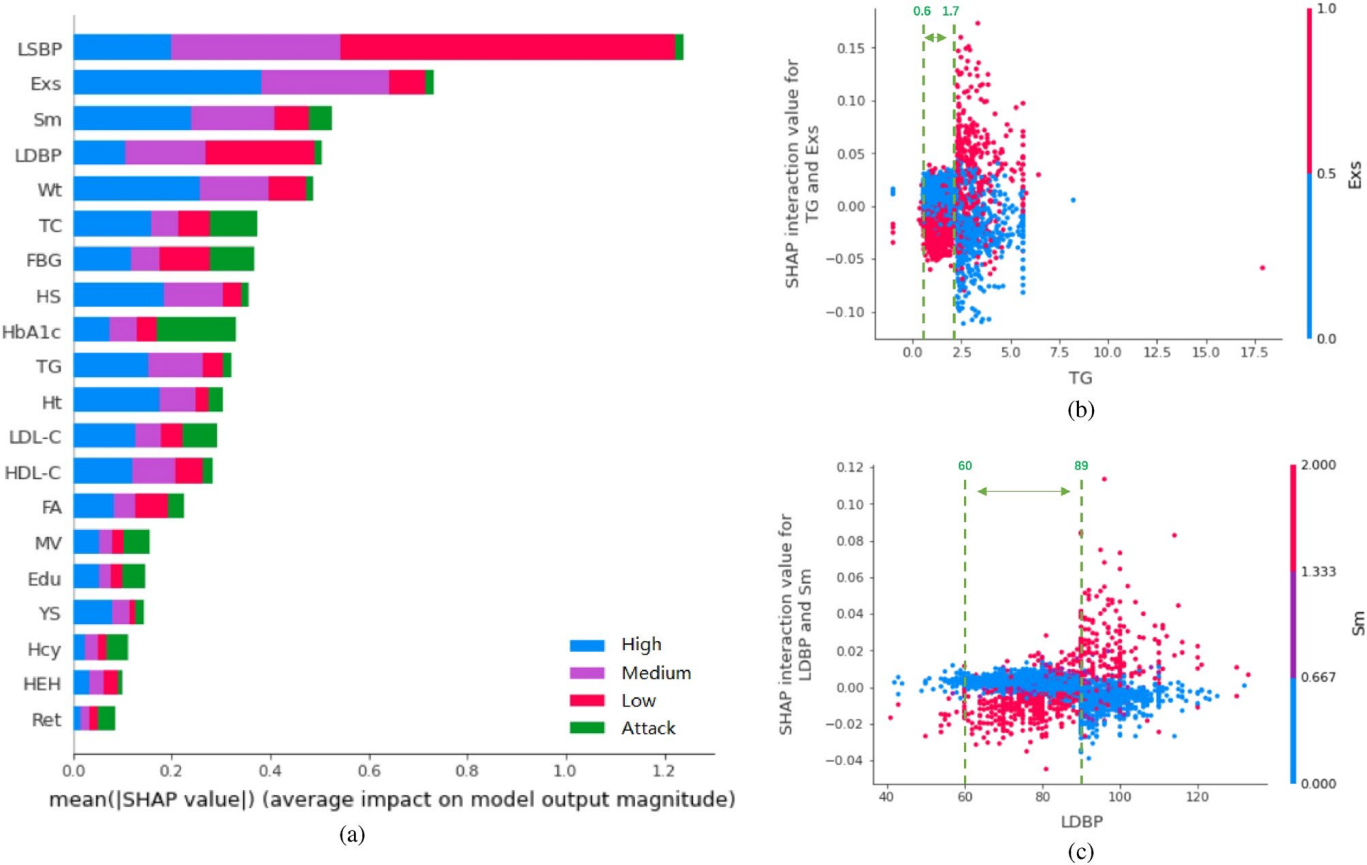
Illustration of Models. (**a**) Ranking of feature selection. (**b**) High-risk explanation: SHAP dependence between Exs and TGs. (**c**) High-risk explanation: SHAP dependence between Sm and LSBP.

According to the average Shapley absolute values of every feature in the samples, the feature importance ranking of the base DNN model can be computed. Figure 3a is the feature importance diagram of the base DNN model. Different from^20^, we provide not only the importance of features in stroke risk prediction but also those for each state. For the four classification model, the features are listed with reduced importance from top to bottom. Different colors in the graph represent the importance of features in different states of prediction. Overall, left systolic blood pressure (LSBP), lack of exercise (Exs) and smoking (Sm) are the top 3 features in stroke risk assessment. Important features for identifying different risk states could also be obtained according to the SHAP values. LSBP, LDBP, FBG, and Exs dominate the importance of low-risk prediction; LSBP, Exs, Sm, and LDBP contribute more to the medium-risk prediction; Exs, Wt, Sm, and LSBP have significant roles in determining the high-risk prediction; while for the prediction of the attack state, HbA1c, TC, LDL-C has a greater marginal effect. The explanations of the selected features are given in Table 1.

### QIDeep: improvement of base DNN model

#### Order-2 feature selection

It has been suggested that there are some synergistic effects between different risk factors^31^. Therefore, to improve accuracy, the interactions between two different features, i.e., order-2 interaction values, are also selected as inputs of the QI layer of QIDeep. The order-2 features are screened by the largest interaction and dependency value calculated by SHAP. By considering both feature importance and interaction strength results of the four states, pairwise interactions among LSBP, Exs, Sm, LDBP, RSBP, HbA1c, and HS are selected as the inputs of the QI layer.

We use the high-risk state explainer as an example. Figure 3b,c describe the interactions of Exs-TG and Sm-LDBP, respectively. The normal ranges of the indicators in Table 1 are also illustrated in these figures (green dotted lines). We note that when the indicators, sorted along the x-axis are not in their normal range, lack of exercise (y = 1) will elevate patients to a high-risk state owing to greater SHAP values. Nonsmoking does not affect high-risk patients, while smoking increases the probability of high-risk patients, especially when blood pressure increases.

#### Numerical results

The improvement results are shown in Table 3. For the QIDeep model, the number of features, shown as 34+N, means that the features are composed of 34 original features and N order-2 interaction features. It can be seen from the table that with an increase in number of order-2 interaction features, the mean of the recall decreased slightly, while the model converges more rapidly by observing the drop in iteration numbers. Specifically, we list the recall value of the attack state for each feature set. It seems that QIDeep with three order-2 interaction features, whose expected recall is 78.81% (95% CI (78.13%, 79.50%)) achieves the best overall performance.

**Table 3:** Influence of The Number of Interaction Features on Performance of QIDeep.

Model	#Features $^{1}$	Mean (R)(%) $^{2}$	R(s) (%) $^{3}$	Mean (P) (%)	Mean (F1 score) (%)	#Iterations $^{4}$
DNN	34	$80.29\pm 0.923$	$78.01\pm 2.162$	$82.10\pm 0.998$	$81.05\pm 0.906$	$48.3\pm 21.34$
QIDeep	34 + 3	$81.16\pm 0.570$	$78.81\pm 2.464$	$83.33\pm 0.674$	$82.02\pm 0.488$	$29.38\pm 13.31$
	34 + 4	$80.63\pm 0.631$	$76.41\pm 2.417$	$83.01\pm 0.706$	$81.57\pm 0.565$	$14.18\pm 5.81$
	34 + 5	$80.23\pm 0.572$	$74.01\pm 2.519$	$82.66\pm 0.794$	$81.19\pm 0.561$	$9.34\pm 3.72$
	34 + 6	$80.05\pm 0.706$	$74.39\pm 2.647$	$82.13\pm 0.582$	$80.87\pm 0.564$	$6.66\pm 1.97$
	34 + 7	$80.15\pm 0.742$	$74.68\pm 2.841$	$82.07\pm 0.722$	$80.91\pm 0.613$	$6.56\pm 2.04$

$^{1}$ #Features: number of features.

$^{2}$ Mean(X): mean of X on all four states, for X $\in \{{\hbox {Precision, Recall, F1 score}}\}$.

$^3$ R(s): recall of the attack state.

$^4$ #Iteration: number of iterations.

**Figure 4:**
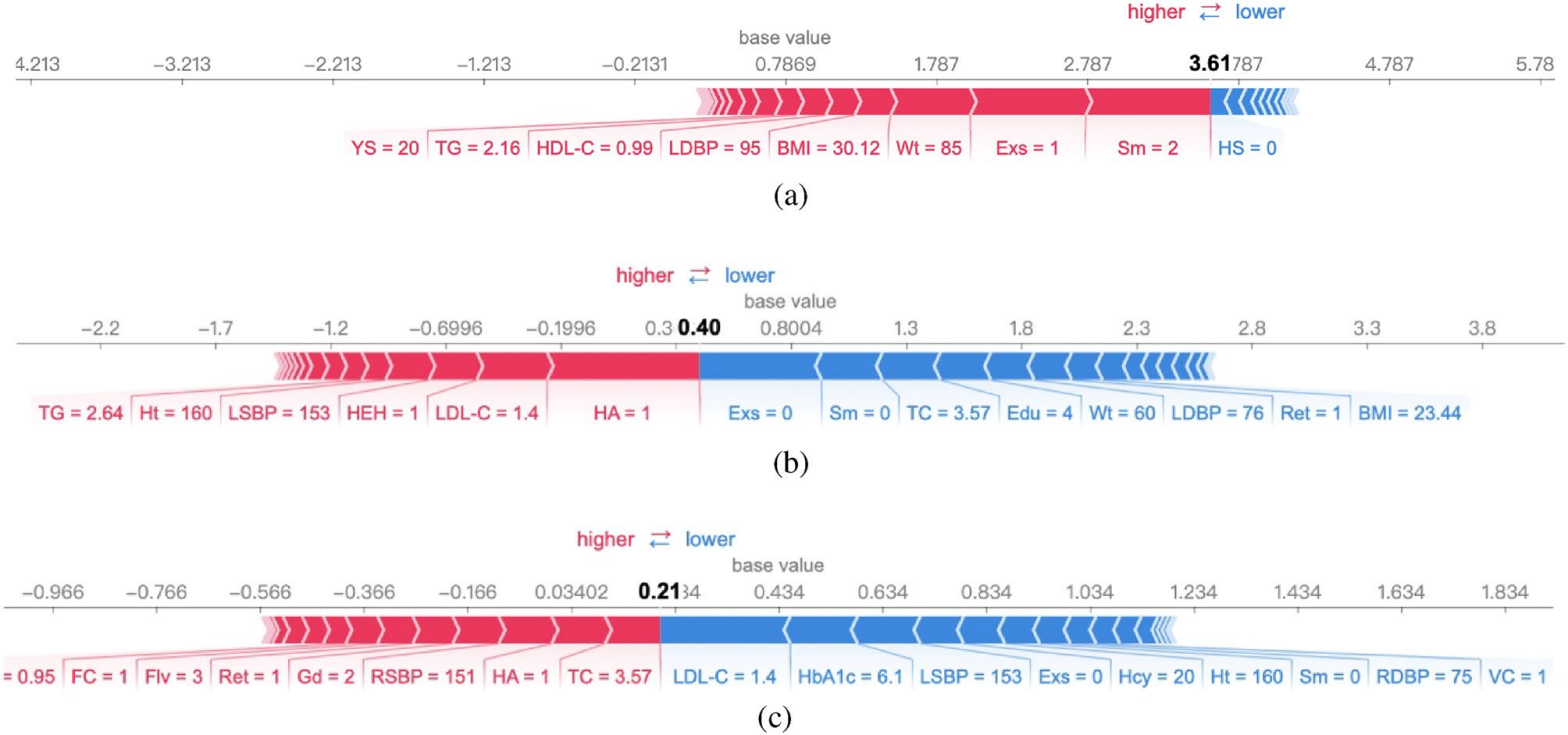
Explanation of two samples correctly predicted to be high-risk. (**a**) Force plot of prediction (0, 0, 3.6051, 0) on high-risk (**b**) Force plot of prediction (0, 0, 0.40, 0.21) on high-risk. (**c**) Force plot of (0, 0, 0.40, 0.21) on attack state.

To increase the interpretability and practicability of the model, SHAP DeepExplainer for the QIDeep model is established to identify the dominant risk factors leading to state transition for each subject. Here we use two examples to explain how SHAP DeepExplainer works.

In the first example, we randomly select a sample of high-risk individuals. The predicted value for the four states is (0,0,3.6051,0), which means that the predicted state is high-risk with a maximum score of 3.6051, and no state transition potential (although according to (2), the predicted value of the four states should sum up to 1, in the numerical experiment, we use the value before *softmax* due to the direct use of the CELoss of PyTorch to avoid a vanishing gradient). Through the single sample analysis tool of SHAP, we obtain four explainers that correspond to the four states. These four explainers take a single sample as input and provide the force graph of each state. Figure 4a shows the force graph obtained by explainers corresponding to the high-risk state. In the figure, Shapley values of feature attributes are visualized as “forces”, and each feature value is a force that increases or decreases the value of the prediction. The prediction starts from the base value, which is the average of the predictions, and each Shapley value is presented as an arrow to increase (red) or decrease (blue) the prediction.

Figure 4a describes that smoking behavior (even worse than 20 years of smoking), lack of exercise, higher BMI, and abnormal indicators, such as blood pressure, elevate this patient to a higher risk state, while no family history of stroke prevents the patient from the high-risk state.

Figure 4b,c is another example of an analysis of high-risk patients. The predictive value for the four states is (0,0,0.40,0.21), which means that the patient tends to change from a high-risk state to an attack state. Combined with Fig. 4b,c, it can be seen that the state is at high-risk due to a family history of stroke and hypertension, current high blood pressure, and older age (Ret = 1). However, the patient does not smoke and continues exercising to ensure a normal BMI and TC, which reduces the risk of stroke. In particular, we can see that the patient has a higher level of education and suggest that better understanding and higher attention to the disease would probably reduce the risk of stroke attack. This finding is consistent with the conclusion of the previous study^2^, i.e., the prevention of stroke can be accomplished by better and earlier treatment of hypertension and health education. Combined with the analysis results, we suggest that this patient should adopt a light diet (Flv) and take drugs to ensure normal blood pressure and cholesterol levels.

### MMOE: proceeding improvement of attack state

#### Model selection for auxiliary objective

Table 4 shows the prediction results of current popular algorithms for stroke occurrence prediction on our dataset. Apart from the abovementioned indicators, the AUC, the area under the ROC curve, is considered to evaluate the performance of binary classifiers in the medical diagnosis domain^32^. The AUC has the role of indicating whether the sorting is correct, i.e., whether the scores of positive samples are greater than those of negative samples because it considers both sensitivity and specificity^33^, and does not depend on the selection of thresholds.

As shown in Table 4, Because DNN-B (B denotes binary to distinguish DNN-B from the base DNN model for four classifications) is the best model with the evaluation of all indicators, it is chosen as Expert1 of MMOE. Compared with LR, gradient harmonizing mechanism logistic regression (GHMLR)^34^ outperforms the imbalanced sample set of stroke occurrence (1:10, as we mentioned) by using gradient density as hedging to disharmonies between different examples. RF is greater than GHMLR as its precision, recall, and f1 score are better with similar AUC values. Therefore, the AUC could not be treated as a unique evaluating indicator in our project.

**Table 4:** Comparison on binary classifications of stroke occurrence prediction.

Model	Precision(%)	Recall(%)	F1 score(%)	Auc
LR	62.03	64.49	63.23	0.8681
GHMLR	78.60	73.11	75.76	0.9395
GBDT	100	53.42	69.64	0.9057
RF	97.92	78.17	86.94	0.9370
DNN-B	93.91	87.46	90.35	**0.9781**

Significant values are in bold.

#### Numerical results

For the MMOE model, the attach occurrence prediction is objective 1, and stroke risk assessment is objective 2. We emphasize that we select the top 20 features from the original 34 features as the input to ensure convergence. The MMOE outperforms the single objective model of both objectives. For attach occurrence prediction, the average recall is increased by 5.68% (from 84.75 to 89.56%), and the AUC is increased by 1.05% (from 97.10 to 98.12%) with similar precision (DNN: 94.94%, MMOE: 94.43%). Consider the stroke risk prediction, the overall prediction accuracy is 84.51% (95% CI (84.17%, 84.84%)) with a feature structure of 20 + 3, which is 1.37% higher than that of the QIDeep model with a feature structure of 20 + 3 (83.14% (95% CI (82.22%, 84.05))) and even 1.18% higher than that of the QIDeep model with a feature structure of 34 + 3 (83.33%). The contents of QIDeep and MMOE of Table 2 show that the prediction performance (precision, recall, and F1 score) of each state is improved by MMOE. We believe that when the dataset is large enough, the two-objective optimization of stroke occurrence and stroke risk prediction based on all features of the MMOE framework would achieve better results.

With the promise of improving the overall effect of the model, we further compare the prediction results of each method for the attack state, as shown in Table 5. This approach is also the motivation for using the multi-objective model. In this case, the base DNN model uses 20 original features, and QIDeep and MMOE use a feature structure of 20 + 3. It can be seen from Table 5 that for the attack state, MMOE is much better than the single objective model (base DNN model and QIDeep model) concerning all the indicators.

For the attack state, compared with the QIDeep model, the F1 score of MMOE is increased by 7.4% (from 78.99 to 84.87%), and the recall is increased by 14.8% (from 71.49 to 82.06%).

**Table 5:** Comparison of attack state prediction results.

Model	Precision(%)	Recall(%)	F1 score(%)
Base DNN	$85.69\pm 1.676$	$70.44\pm 3.712$	$77.24\pm 2.119$
QIDeep	${\mathbf {88.41}\pm 3.094}$	$71.49\pm 1.875$	$78.99\pm 0.070$
MMOE	$87.97\pm 2.056$	${\mathbf {82.06}\pm 3.043}$	${\mathbf {84.87}\pm 1.944}$

Significant values are in bold.

### Managerial implication

Traditionally, laboratory data is collected by the doctor during the diagnosis. The stroke risk state is estimated according to the doctor’s personal experience along with the 8+2 rules from the CSPP. Generally, the doctors cannot attribute the risk state to more detailed factors. This paper aims to approach personalized and intelligence diagnostics. Through accuracy prediction models and their explainers, the proposed methods produce the main factors affecting the current risk state and the transfer trends for each patient individually. The returns of the proposed methods can help the doctors to diagnose, and be demonstrated visually to the patients for a clear sense of the factors and the actions suggested to react, such as quitting smoking and exercising.

## Conclusion

In view of the diagnosis and analysis of stroke, stroke risk assessment models have been proposed by deep learning methods. Moreover, the proposed models could identify the determinants of these risk states for every subject, which makes personalized treatment possible and improves the effectiveness of stroke intervention and prevention.

Specifically, for the accuracy of all stroke risk predictions, we propose a QIDeep model by adding quadratic interactive features to a DNN to address a small data set. This method can flexibly control the number of combination features, and balance model parameters and samples to ensure model convergence. In addition, aimed at the problem that the method is not effective for the important attack state, we use the multi-objective learning framework of MMOE for reference and build a model with the objective of optimizing both stroke occurrence prediction and stroke risk prediction. In the case of the same feature, the prediction effect of each risk state is improved, the accuracy of the stroke risk assessment was improved by 1.37% (from 83.14 to 84.51%), and the F1 score of the attack state was increased by 7.4% (from 78.99 to 84.87%) and the recall is increased by 14.8% (from 71.49 to 82.06%) compared with QIDeep of the single objective method. This method can be applied to the prediction of other diseases, where missing data are common and risk factors are not well understood.

Unfortunately, SHAP estimates the feature importance by linear approximation. The explainer can order the features by their attributions but the attributions are not precisely quantified. Hence, its application to precision medicine is not straightforward. In the future, we are going to improve the performance of stroke risk assessment by collecting data from multiple dimensions. To best benefit from the census data, we will try the meta-learning framework^35^. More precisely, the meta-knowledge of the intelligence diagnostics will be trained on the lifestyle data, while turning to the specific disease. The goal is to achieve few-shot convergence and accurate prediction on the small laboratory dataset.

## Data Availability

The implementation of the numerical experiment is available at https://github.com/MadelineMa/MTL4StrokeAssessment. Despite the application to the stroke dataset, we also apply the models to the MNIST handwritten digit dataset (https://github.com/ MadelineMa/MTL4StrokeAssessment/tree/main/src/MNIST), which offers evidence of the generalization of the proposed methods.
